# Effects of Binge Drinking and Alcohol Withdrawal on Serum Neurofilament Levels

**DOI:** 10.1111/adb.70183

**Published:** 2026-09-15

**Authors:** Ann‐Kathrin Stock, Maximilian Pilhatsch, Katja Akgün, Joris C. Verster, Elisabeth Brückner, Mona Hofmann, Julian R. Hinz, Dušan A. Ladjević, Tjalf Ziemssen, Christian Beste

**Affiliations:** ^1^ Cognitive Neurophysiology, Department of Child and Adolescent Psychiatry, Faculty of Medicine TU Dresden Dresden Germany; ^2^ Department of Psychiatry and Psychotherapy Carl Gustav Carus University Hospital, TU Dresden Dresden Germany; ^3^ Department of Psychiatry and Psychotherapy ELBLANDKLINIKUM Radebeul Germany; ^4^ Center of Clinical Neuroscience, Department of Neurology University Hospital Carl Gustav Carus, Technical University of Dresden Dresden Germany; ^5^ Division of Pharmacology, Utrecht Institute for Pharmaceutical Sciences Utrecht University Utrecht the Netherlands; ^6^ Centre for Mental Health and Brain Sciences Swinburne University Melbourne VIC Australia; ^7^ Yale Child Study Center Yale University New Haven Connecticut United States; ^8^ Department of Behavioral Sciences Oslo Metropolitan University Oslo Norway

**Keywords:** alcohol, binge drinking, cognition, neurofilament light chain, white matter, withdrawal

## Abstract

Chronic heavy alcohol use and alcohol withdrawal are known to diffusely damage white matter (WM), but it has remained rather unclear whether the damage sustained during a single withdrawal event is functionally relevant and whether WM damage also occurs during or after single episodes of heavy drinking. We therefore set out to investigate the effects of acute alcohol intoxication, alcohol hangover and (post)acute alcohol withdrawal on axonal injury (assessed via serum neurofilament light chain/NFL) and potentially associated cognitive‐behavioural functioning (as assessed with the CANTAB test battery) in three separate studies. Acute intoxication and hangover effects were studied in healthy young men who underwent a standardized intoxication procedure aiming at an intoxication level of approx. 1.2‰ in two separate randomized experimental crossover studies. (Post)acute withdrawal effects were longitudinally observed in withdrawal patients during a 21‐day inpatient qualified detoxification treatment. There was no significant effect of alcohol intoxication (*n* = 51) or alcohol hangover (*n* = 80) on NFL levels. In contrast, withdrawal patients (*n* = 31) revealed significant changes of NFL levels over the course of inpatient treatment; most prominently an increase from the first day of admission to the peak of withdrawal symptoms and a decrease from there to the day of hospital discharge. Higher NFL levels of withdrawal patients were associated with slower motor responses and lower risk taking/delay aversion in gambling, as assessed with the CANTAB test battery. In conclusion, the functionally relevant increase in axonal injury despite gold‐standard inpatient treatment for alcohol withdrawal necessitates further investigation.

## Graphical abstract

In n=31 alcohol use disorder patients seeking inpatient qualified detoxification, the study found a significant increase in serum neurofilament (NFL) levels from hospital admission (T1; day 1) to active withdrawal (T2; days 3−4), which slightly decreased after active withdrawal (T3; days 5−7), and further significantly decreased again until hospital discharge (T4; day 21).

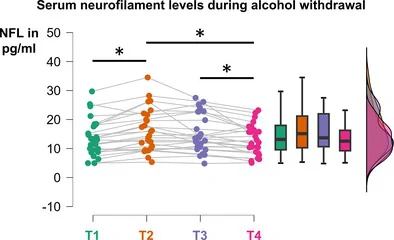

Not depicted: No significant changes in NFL levels were found in two samples of healthy young men during experimentally induced alcohol intoxication (n = 51) and hangover (n = 80).

## 1 Introduction

Harmful alcohol use is one of the largest risk factors for disease, disability and death worldwide [1]. Although the data on moderate alcohol consumption are somewhat less conclusive [2], excessive alcohol consumption, especially in the context of alcohol use disorder (AUD), is clearly associated with cognitive decline and dysfunction, most frequently in executive functioning and self‐control [3, 4]. These consequences are typically attributed to neuronal degeneration as a result of alcohol neurotoxicity affecting both grey matter (GM) and white matter (WM) [4]. It has repeatedly been demonstrated that excessive alcohol consumption is associated with GM reductions in functionally relevant brain areas such as the caudate nucleus [5] prefrontal cortex, anterior cingulate cortex and hippocampus [6, 7]. Recent studies further suggested that deficits in connectivity and WM microstructure also likely play a key role in many of the relevant functional deficits [8, 9]. Our study specifically set out to focus on the latter.

Moderate‐to‐high alcohol consumption has been shown to result in widespread diffuse damage to the adult brain, especially with respect to WM microstructure [9, 10]. These changes are linked to alcohol‐induced cognitive dysfunction and, as a result, increasing difficulties in achieving or maintaining abstinence [10, 11]. Despite the fact that ‘no level of alcohol consumption is safe for our health’ [12] and clear evidence that sufficient ethanol concentrations or excessive consumption habits are detrimental to the survival and function of cells in the adult brain (with a linear relationship between the consumed amount of alcohol and associated brain changes as assessed with MRI [13]), there is no consensus on appropriate or ‘gold‐standard’ diagnostic markers of alcohol‐related neuronal damage, especially to WM, in humans. Diffusion tensor imaging (DTI) has been demonstrated to detect demyelination‐associated changes in AUD [10, 14] but is most likely not sensitive enough to reliably detect changes induced by a single drinking incident. Therefore, it has remained unclear whether a single dose/binge drinking event already induces relevant WM brain damage in otherwise healthy drinkers [10]. Single molecule array (SIMOA) analyses were recently shown to be highly sensitive at detecting axonal injury/changes in WM microstructure (and, albeit to a lesser degree, GM) via the quantification of neurofilaments (NFL) [15, 16, 17, 18, 19, 20]. NFL are microstructural proteins that are found in neurons and released into the cerebrospinal fluid as well as the bloodstream in case of axonal damage/degeneration. They have successfully been demonstrated to strongly correlate with DTI‐detected WM changes and to reliably indicate changes in several WM‐associated pathologies, including multiple sclerosis, dementia or traumatic brain injury [21, 22, 23, 24]. Serum NFL could therefore be a promising potential marker of alcohol‐induced axonal injury as well as a potential correlate of symptom severity and associated cognitive decline [25, 26, 27, 28] and will thus be used as such in this study.

For the purpose of this study, we will base our hypotheses on the assumption that glutamatergic excitotoxicity (rather than direct alcohol toxicity) could be one of the main drivers of alcohol‐induced axonal injury/WM damage. The underlying reasoning is as follows: Individuals with moderate‐to‐severe AUD do not only engage in regular alcohol binges but often maintain a rather steady baseline level of alcohol consumption. This leads to tolerance development with homeostatic adaptations in the regulation of many vital parameters (e.g., heart and respiratory rate and body temperature) as well as neuroadaptive processes, including changes in the synthesis and synaptic binding of important neurotransmitters (especially glutamate and GABA) [29, 30]. These counterregulatory mechanisms, including the increased synthesis of glutamate and the decreased synthesis of GABA [30], may cause life‐threatening complications (e.g., cardiovascular emergencies, respiratory problems, inflammation, fever, hallucinations or seizures) when abstinence is initiated. Although vital functions of withdrawing patients are closely monitored in every clinic, rather little attention is currently paid to the underlying neuroadaptive mechanisms, especially to the fact that tolerance‐associated GABAergic and glutamatergic dysregulation [30] likely results in neuronal hyperexcitability during withdrawal [29]. The common first‐line treatment with positive GABA modulators like benzodiazepines [31] provides effective symptom relief and protection against the increased risk of epileptic seizures [32] but largely ignores the fact that aberrant glutamatergic signalling is a major risk factor for excitotoxic neural damage [33] that may not be sufficiently addressed by benzodiazepine administration alone. Even though epilepsy and excitotoxicity share underlying neurobiochemical mechanisms and alcohol‐induced glutamatergic neural damage has been demonstrated in rodents both in vitro and in vivo [33, 34], glutamatergic excitotoxicity does not seem to have been investigated as a WM pathology‐driving mechanism in withdrawing human subjects, let alone widely acknowledged in clinical practice. If glutamatergic neurotoxicity was however the cause of the withdrawal‐associated WM damage demonstrated in previous studies [9, 10, 14], this would be of paramount importance for the future clinical treatment of alcohol withdrawal and might necessitate increases in the frequency, dosage and duration of anticonvulsant administration and/or the administration of NMDA receptor antagonists [35] in order to minimize neuronal damage.

Based on the assumption that glutamatergic excitotoxicity drives alcohol‐induced axonal injury/WM damage, we hypothesized that NFL should only be elevated during phases of acute and chronic tolerance (i.e., mainly in the absence of alcohol intoxication), thereby showcasing that alcohol tolerance, and not alcohol itself drives axonal injury. Based on this, we hypothesized that serum levels of NFL should:

1 Not be elevated during acute alcohol intoxication (as compared to the sober state). This was assessed in Study 1.
2 Be (slightly) elevated during acute alcohol hangover, which by definition starts once alcohol can no longer be detected in blood or breath [36] (as compared to the sober nonhungover state). This was assessed in Study 2.
3 Be significantly elevated during acute alcohol withdrawal and decrease over the course of inpatient treatment, as the tolerance slowly wears off. This was assessed in Study 3.

If, however, alcohol intoxication itself caused the majority of WM damage, we would expect to see similar increases in NFL in both intoxication (Study 1) and hangover (Study 2), whereas there should be no increase with withdrawal symptoms, but rather a steady decrease in NFL levels with protracted abstinence/time in the clinic (Study 3).

Given that previous studies suggested a functional link between NFL levels and cognitive performance [16, 19, 37, 38], we further hypothesized that:

4 Serum levels of NFL should be inversely correlated with cognitive performance (as assessed with the CANTAB battery). This was separately assessed in all three study samples.

## 2 Subjects and Methods

### 2.1 Study Design

**FIGURE 1:**
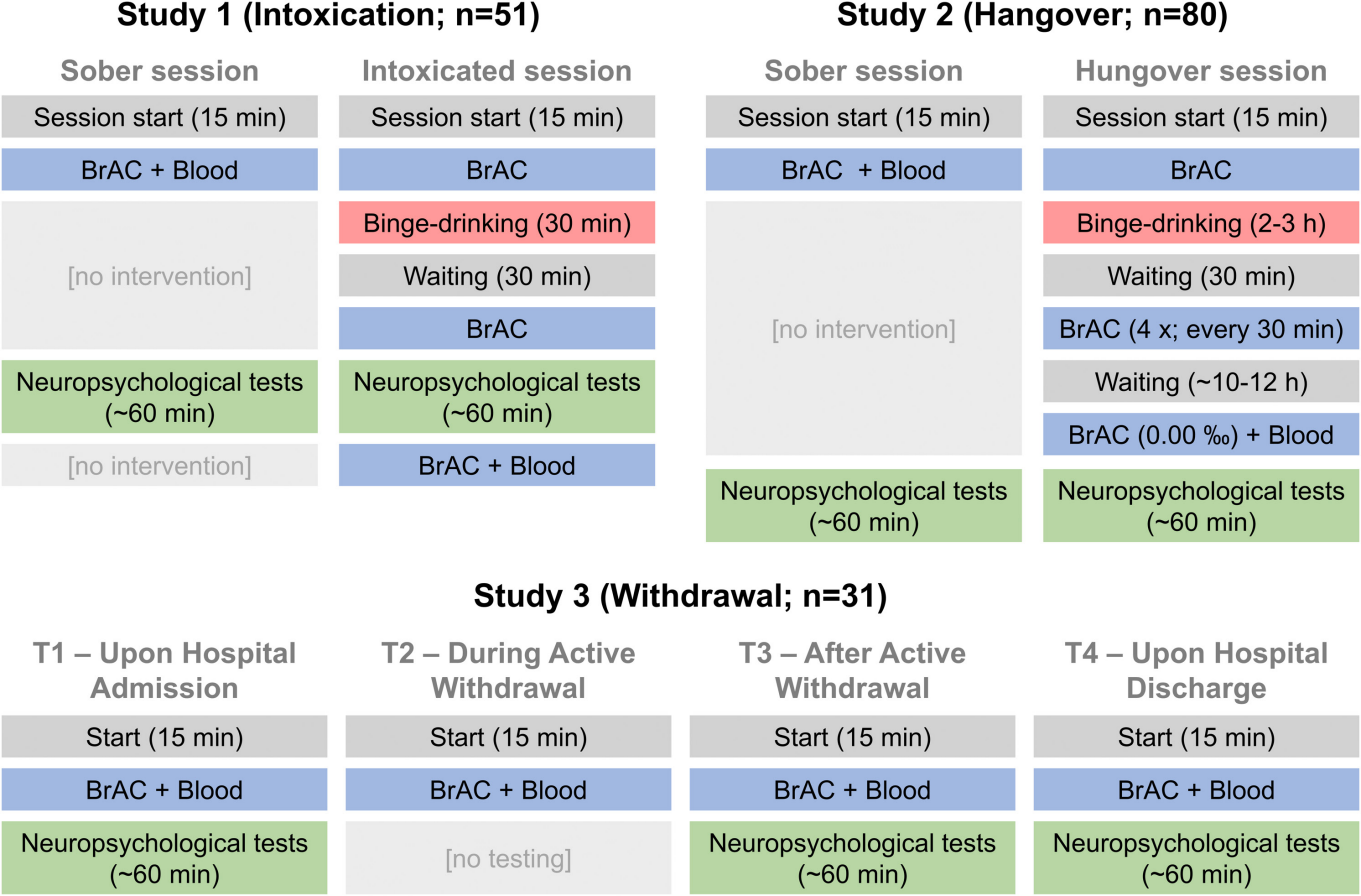
Illustration of the three different studies reported in this paper. Studies 1 and 2 were experimental, and healthy participants had to binge‐drink on one of the appointments (red box), which varied in order across the study samples (top). Study 3 was observational and all AUD patients underwent the appointments in the same order (bottom). BrAC = Breath Alcohol Concentration; Neuropsychological Tests refer to the CANTAB tasks described in the methods section and supplement. The sample sizes provided for each study (*n*) refer to the number of study participants included in the final neurofilament analysis (please note that the number of cases with CANTAB assessments is lower in Studies 1 and 2, see Table 1). Further details on each study appointment and alcohol administration can be found in the Supporting Information.

We assessed acute alcohol intoxication effects, acute alcohol hangover effects and acute alcohol withdrawal effects in three separate data collections/studies (illustrated in Figure 1).

For the assessment of acute intoxication effects (Study 1) and of acute alcohol hangover effects (Study 2) in healthy young men, we chose randomized interventional/experimental crossover designs, for which each participant was assessed once while sober (both Studies 1 and 2), and once while acutely intoxicated in an experimental/standardized manner (Study 1), or while acutely hungover after a night of standardized experimentally induced intoxication (Study 2). In both Study 1 and Study 2, we aimed for an experimental intoxication of around ~1.2‰ based on the Widmark‐Watson equation [39, 40]. Of note, some of the healthy individuals participated in both Study 1 and Study 2 on separate occasions, thus leading to a partial overlapping of samples between Studies 1 and 2. The data from these two studies were however still collected and analysed separately.

For the assessment of acute withdrawal effects (Study 3) in clinical patients, we conducted a noninterventional/observational longitudinal design, for which each participant was assessed once at the beginning of their inpatient withdrawal treatment (T1/first day at the hospital), once at the maximum of their physical withdrawal symptoms (T2/usually on days 3–4 of the hospital stay), once after the physical withdrawal symptoms had subsided (T3/usually on days 5–7 of the hospital stay) and once before they were discharged from the clinics where they received treatment (T4/usually the 21st day of their hospital stay).

For further details on the experimental design, please refer to Figure 1 and the Supporting Information.

### 2.2 Samples

For the acute intoxication and hangover studies, healthy male participants aged 18–31 were recruited via online advertisements, flyers and posters. For the acute withdrawal study, participants seeking inpatient detoxification from AUD aged 18–65 were recruited by the medical staff of the University Clinics Dresden and the ELBLANDKLINIKUM Radebeul when they applied for inpatient withdrawal treatment. Patients were treated in accordance with the national guidelines for the treatment of alcohol‐related disorders [31]. The full eligibility criteria and sample size estimation are described in Supporting Information.

### 2.3 Experimental Alcohol Administration (Studies 1 and 2)

The experimental administration of a standardized amount of alcohol followed the same protocol as described in Opitz et al. [41]. In short, we aimed to achieve a maximum intoxication level of approximately 1.2‰ by administrating an individually determined amount of vodka and orange juice on an empty stomach in Study 1 and an individually determined amount of brandy and/or red wine in Study 2. In Study 1, participants were tested during acute alcohol intoxication (see Figure 1) whereas in Study 2, participants were tested the following day after regaining full sobriety, as defined by a breath alcohol concentration (BrAC) of 0.00‰. For further details on the intoxication procedure, please refer to the Supporting Information.

### 2.4 Quantification of NFL and Thiamine Concentrations

For the participants in Study 3, vitamin B1 (thiamine) was quantified from the T1 EDTA blood samples on the day of sampling by the clinical laboratory of the University Clinics Dresden. The reference range provided by the laboratory was 66.5–200.0 nmol/L.

The serum samples for NFL quantification in all three studies were stored at −26°C till measurement. sNfL measurement was performed using the Advantage NF‐Light singleplex Kit and prepared as defined in the manufacturer's instructions (Quartered, Lexington, MA, Datasheet Quanterix: Simoa NF‐Light Advantage Kit). The Simoa Human Advantage NfL assay is a digital immunoassay for the quantitative determination of NfL and was ran on a Simoa HD‐1 instrument (Quanterix) [42, 43]. Samples were prepared as recommended in the manufacturer's instructions (Quartered, Lexington, MA, USA). Sample dilution was calculated and done by the instrument. Both the mean intraassay coefficient of variation of duplicates and the mean interassay coefficient of variation were < 10%.

### 2.5 Questionnaires

At the beginning of their respective intoxication appointment (and thus before alcohol administration), all participants in Study 1 and Study 2 provided information on height, weight and sociodemographic characteristics and filled in the Beck Depression Inventory (BDI) [44, 45] to determine potential depressive symptoms. They also filled in the Alcohol Use Disorders Identification Test (AUDIT) [46]. In Study 2, participants additionally rated the overall severity their hangover symptoms on an 11‐point Likert scale, which reached from 0 (nonexistent) to 10 (extreme). Patients in Study 3 filled in sociodemographic questionnaires, as well as the AUDIT and BDI on their T3 and T4 appointments in order to minimize the strain on patients during early stages of withdrawal (i.e., during T1 and T2).

### 2.6 Experimental Paradigm

Part of the participants in Study 1 (intoxication) and Study 2 (hangover), as well as all of the participants in Study 3 (withdrawal) were asked to complete several cognitive tests from the Cambridge Neuropsychological Test Automated Battery (CANTAB; Cambridge Cognition Ltd), which had been chosen to most likely reflect alcohol‐related cognitive deficits. From the domain of ‘psychomotor speed’, this included the Reaction Time test (RTI). From the domain of ‘attention’, this included the Rapid Visual Information Processing test (RVP). From the domain of ‘executive function’, this included the Spatial Working Memory test (SWM), the Stop Signal Task (SST), the Cambridge Gambling Task (CGT) and the Intraextra Dimensional Set Shift task (IED). Detailed descriptions of the analysed parameters can be found in the Supporting Information.

Due to typically large learning effects, the IED was only assessed once (on the intoxicated appointment in Study 1, on the hungover appointment in Study 2 and on the T4 appointment in study 3). All other tests were performed at all appointments at which CANTAB performance was assessed (sober and intoxicated appointment in Study 1, sober and hungover appointment in Study 2 and T1/T3/T4 in Study 3, see Figure 1).

### 2.7 Statistical Analyses

Statistical analyses were run with JASP (version 0.17.1).

Hypothesis 1 was investigated by analysing the data collected in Study 1. Specifically, we conducted undirected independent sample *t*‐tests/Mann–Whitney *U* tests to explore the possible effects of appointment order and (un)directed paired sample *t*‐tests/Wilcoxon signed‐rank tests as well as add‐on Bayesian tests to assess whether NFL levels in the sober control condition differed from those during acute alcohol intoxication.

Hypothesis 2 was investigated by separately analysing the data collected in Study 2. Specifically, we conducted undirected independent sample *t*‐tests/Mann–Whitney *U* tests to explore the possible effects of appointment order and (un)directed paired sample *t*‐tests/Wilcoxon signed‐rank tests as well as add‐on Bayesian tests to assess whether NFL levels in the sober control condition differed from those during acute alcohol hangover.

Hypothesis 3 was investigated by separately analysing the data collected in Study 3. Specifically, we conducted a repeated‐measures ANOVA with time (T1 vs. T2 vs. T3 vs. T4) as within‐subject factor. In case of significant differences, adjusted post hoc paired sample *t*‐tests (parametric and Bayesian) were run.

For Hypothesis 4, we conducted exploratory Pearson correlation tests relating NFL levels to selected behavioural measures (as well as the number of diagnostic AUD criteria, the AUDIT score and a few selected sociodemographic measures including age, height and weight). This was done separately for the data from Studies 1, 2 and 3.

For significant appointment comparisons (as well as for statistical trends towards significance), the mean difference plus a 95% confidence interval (CI) as well as a measures of effect size are provided. Descriptive data are reported as mean ± standard deviation (SD).

## 3 Results

### 3.1 Sample Characteristics

Descriptive sample characteristics for the participants included in each of the three studies are provided in Table 1. Detailed information on exclusions and missing data for each study can be found in the Supporting Information.

**TABLE 1:** Descriptive sample characteristics of each study.

Measure	Study 1 (Intoxication)	Study 2 (Hangover)	Study 3 (Withdrawal)
General characteristics			
Analysed sample size	*n* = 51 for NFL	*n* = 80 for NFL	*n* = 31 for NFL
	*n* = 24 for CANTAB	*n* = 35 for CANTAB	*n* = 31 for CANTAB
Sex	*n* = 51 ♂ for NFL	*n* = 80 ♂ for NFL	*n* = 23 ♂ for both
	*n* = 24 ♂ for CANTAB	*n* = 80 ♂ for CANTAB	*n* = 8 ♀ for both
Age in years	22.8 ± 3.9	22.7 ± 3.0	47.5 ± 8.8
Height in cm	182.6 ± 7.0	182.7 ± 6.4	168.8 ± 33.5
Weight in kg	77.3 ± 9.6	78.2 ± 9.6	75.3 ± 15.6
BDI score	2.8 ± 3.2	2.9 ± 2.8	14.1 ± 8.5
Alcohol‐related measures			
AUDIT score	7.1 ± 3.1	8.8 ± 2.8	27.3 ± 5.7
Number of diagnostic AUD criteria according to DSM‐V	*Not assessed*	*Not assessed*	7.6 ± 2.4
Administered alcohol in gram	90.8 ± 7.1	121.8 ± 9.5	*No administration of alcohol as part of study participation*
Highest BrAC measured during study participation in per mille	1.13 ± 0.18	1.24 ± 0.21	0.98 ± 1.01
Overall hangover severity rating (on a scale of 0–10)	*Not assessed*	3.66 ± 2.10	*Not assessed*
Serum thiamine concentration upon hospital admission in nmol/L	*Not assessed*	*Not assessed*	130.1 ± 36.6 range 76–188

*Note:* Please note that for Studies 1 and 2, only a subset of all participants performed the CANTAB test battery (see analysed sample size).

Abbreviations: AUD = Alcohol Use Disorder; AUDIT = Alcohol Use Disorders Identification Test; BDI = Beck Depression Inventory; BrAC = Breath Alcohol Concentration.

### 3.2 Acute Alcohol Intoxication Effects on NFL Levels (Hypothesis 1/Study 1)

In study 1, *n* = 25 participants were intoxicated on their first study appointment and *n* = 26 participants were intoxicated on their second study appointment. These two groups did not significantly differ in terms of the amount of experimentally administered alcohol, maximal BrAC and sober or intoxicated NFL levels (all *p* ≥ 0.158; BF_01_ ≥ 1.538).

**FIGURE 2:**
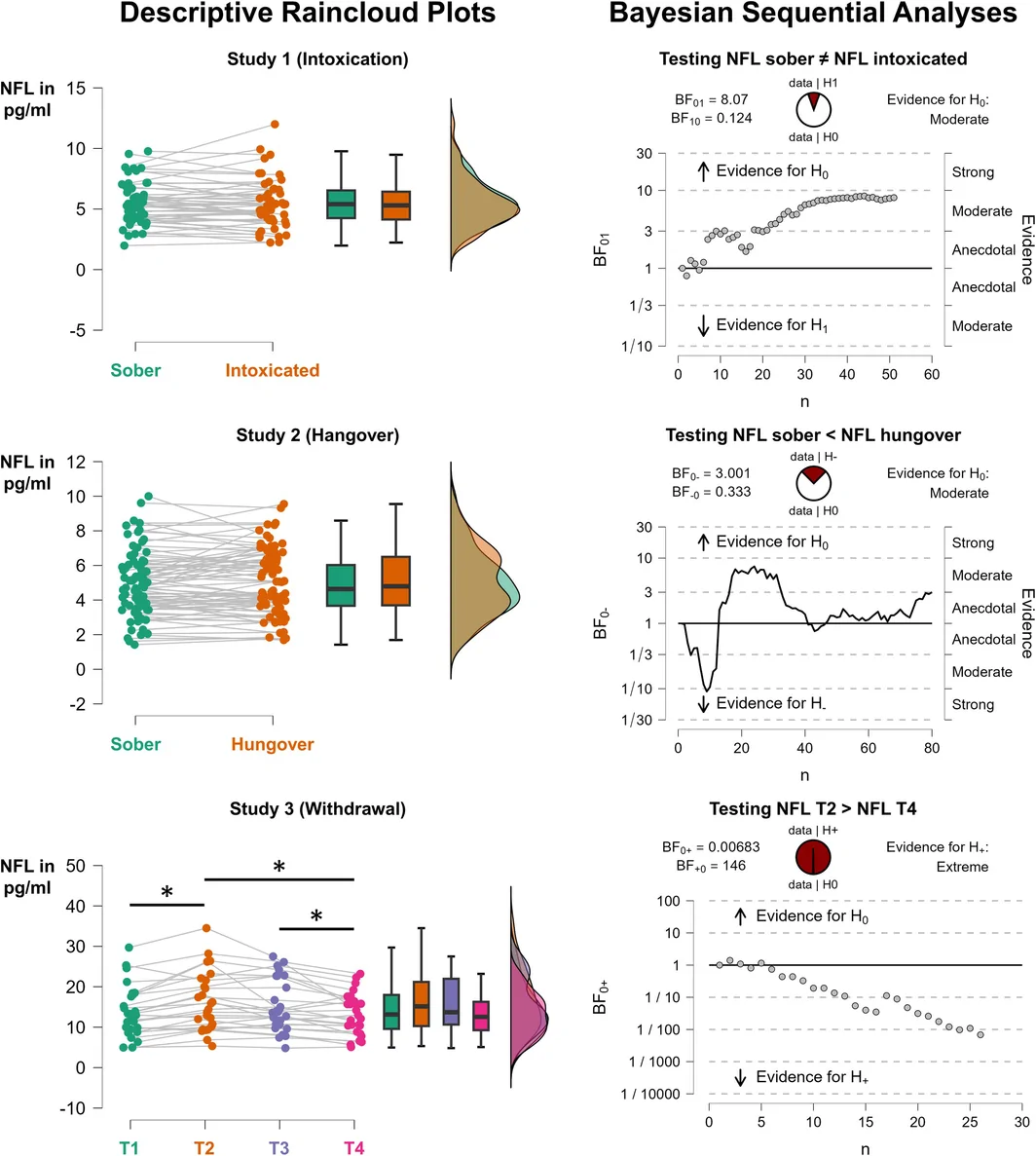
Results of the NFL analyses (which were separately/independently conducted for each study). Descriptive data for the appointments of each study is depicted in the raincloud plots in the left column. Bayesian sequential analyses are illustrated in the right column. In Study 1, there was no significant difference between sober and intoxicated NFL levels, which was supported by moderate Bayesian evidence for the null hypothesis. In Study 2, NFL levels were not significantly larger during the hangover appointment than during the sober appointment, which was supported by moderate Bayesian evidence for the null hypothesis. In Study 3, there was a significant increase in NFL levels from admission (T1) to active withdrawal (T2), which significantly decreased again until the day before hospital discharge (T4). The latter was substantiated by extreme Bayesian evidence for this effect.

When comparing the NFL levels of the sober and intoxicated study appointments (undirected Wilcoxon signed‐rank test), there was no significant difference (*p* = 0.279) between the NFL levels of the sober appointment (5.506 nmol/L ± 1.762) and the intoxicated appointment (5.436 nmol/L ± 2.028). Add‐on Bayesian tests provided moderate evidence for the assumption that NFL levels did indeed not differ between the sober and the intoxicated state (BF_01_ = 8.07). Hence, our results confirmed Hypothesis 1. A graphical representation of the results and a sequential analysis plot are provided in Figure 2.

### 3.3 Acute Alcohol Hangover Effects on NFL Levels (Hypothesis 2)

In study 2, *n* = 37 participants were hungover on their first study appointment and *n* = 43 participants were hungover on their second study appointment. These two groups did not significantly differ in terms of the amount of experimentally administered alcohol, maximal BrAC, overall hangover severity rating and sober or intoxicated NFL levels (all *p* ≥ 0.061; BF_01_ ≥ 0.929).

When comparing the NFL levels of the sober and hungover study appointments (directed paired samples *t*‐test assuming larger NFL values in the hungover condition), there was a trend (−0.126 nmol/L; 95% CI ‐∞ to 0.043; *p* = 0.109; *d*' = −0.139) between the NFL levels of the sober appointment (4.906 nmol/L ± 1.870) and the hungover appointment (5.032 nmol/L ± 1.910). Yet, add‐on Bayesian tests provided moderate evidence for the assumption that NFL levels did not differ between the sober and the hungover state (BF_01_ = 3.00). Hence, our results did not sufficiently substantiate Hypothesis 2. A graphical representation of the results and a sequential analysis plot are provided in Figure 2.

### 3.4 Alcohol Withdrawal Effects on NFL Levels (Hypothesis 3)

In Study 3, the repeated‐measures ANOVA revealed a main effect of time point (*p* < 0.001; partial *η* ^2^ = 0.289). Adjusted post hoc *t*‐tests demonstrated that NFL levels significantly increased from the first day of hospital admission (T1: 13.918 nmol/L ± 6.340) to the day when withdrawal symptoms were assumed to be maximal (T2: 16.338 nmol/L ± 7.325) (2.421 nmol/L; 95% CI 0.719 to 4.122; *p* = 0.001; *d'* = −0.379). We further found that NFL levels on the day before discharge from the clinic were significantly lower (T4: 13.156 nmol/L ± 5.190) than during withdrawal (T2) (−3.182 nmol/L; 95% CI −4.884 to −1.481; *p* < 0.001; *d*' = 0.498) and then after withdrawal (T3: 15.265 nmol/L ± 6.508) (−2.109 nmol/L; 95% CI −3.810 to −0.407; *p* = 0.005; *d*' = 0.330). The nonsignificant differences between T1 and T3 (*p* = 0.105; BF_01_ = 4.237), as well as T1 and T4 (*p* = 0.229; BF_01_ = 3.836) were substantiated by add‐on Bayesian testing providing moderate evidence that there was no difference. For the nonsignificant difference between T2 and T3 add‐on Bayesian testing provided anecdotal evidence for the null hypotheses (*p* = 0.183; BF_01_ = 0.714). For the decrease from T2 to T4 (assessed exemplarily), Bayesian evidence provided extreme evidence (BF_01_ = 0.007). A graphical representation of the results is provided in Figure 2.

### 3.5 Associations Between NFL Levels and Cognitive‐Behavioural Performance (Hypothesis 4)

Because we found no significant effects of alcohol intoxication on NFL in Study 1, we only correlated the sober NFL values and AUDIT scores to the sober CANTAB task performance variables detailed in the Supporting Information. These analyses did not result in a functionally relevant pattern of correlations between NFL and the assessed cognitive measures, as the only significant finding in Study 1 was a negative correlation of NFL levels with the number of overall errors in the RTI. All other correlations between NFL levels and the assessed CANTAB variables remained nonsignificant. There were however a few correlations suggesting that participants with higher AUDIT scores were inclined to respond faster and more correctly in the RTI while also having faster visual processing in the RVP (please refer to the Supporting Information for more details).

Because we found no significant effects of alcohol hangover on NFL in Study 2, we only correlated the sober NFL values and AUDIT scores to the sober CANTAB task performance variables detailed in the Supporting Information. These analyses did not result in a functionally relevant pattern of correlations between NFL and the assessed cognitive measures, as the only significant finding in Study 2 was a positive correlation of NFL levels with the number of completed blocks in the CGT. All other correlations between NFL levels and the assessed CANTAB variables remained nonsignificant. There were however a few correlations suggesting that participants with higher AUDIT scores were inclined to respond more accurately in the RTI and have slightly worse spatial memory in the SWM (please refer to the Supporting Information for more details).

Next, we correlated the NFL levels obtained in T1, T2, T3 and T4 of Study 3, as well as the BrAC and serum thiamine levels at T1, AUDIT scores and the number of fulfilled diagnostic DSM‐V AUD criteria with the CANTAB performance levels separately for each of the three study appointments. Apart from the intercorrelation of NFL values assessed at the different time points of Study 3, we found a positive correlation between BrAC levels upon hospital admission (at T1) and NFL levels upon hospital discharge (at T4), as well as positive correlations between the AUDIT score and NFL levels upon hospital admission (at T1), between the AUDIT score and NFL levels during active withdrawal (at T2) and between the AUDIT score and the number of DSM‐V diagnostic criteria for AUD. The most consistent findings were that higher BrAC values/lower thiamine levels upon admission (at T1) were associated with slower response times/motor responses in the RTI and RVP and poorer decision making in gambling in the CGT. Higher NFL levels were associated with slower response times/motor responses (including the stop signal reaction time) in the RTI and SST as well as with lower risk taking and delay aversion in gambling in the CGT on the respective assessment days (please refer to the Supporting Information for more details).

Overall, NFL values mainly correlated with cognitive performance in withdrawing patients (Study 3), but not with (sober) performance in healthy young men (both Study 1 and Study 2). For further details (including *r*‐, *p*‐ and *z*‐values) on the correlations found in Study 3, please refer to the Supporting Information.

## 4 Discussion

In summary, our results show that neither a binge‐like alcohol intoxication nor a medium‐severity alcohol hangover are associated with detectable axonal degeneration/WM damage in otherwise healthy young men. These findings refute the ideas that a binge‐like intoxication or acute tolerance in the context of alcohol hangover cause measurable/relevant axonal injury/WM damage. Likewise, we did not observe a significant correlation between drinking habits within the past 12 months (as reflected by the AUDIT score) and NFL levels in those two samples (see Supporting Information). This refutes the idea that drinking patterns as observed in healthy young men who do not fulfil the diagnostic criteria for AUD directly translate to significant axonal injury/WM damage as reflected by NFL.

In contrast to this and confirming a previous DTI‐based report by colleagues [14] as well as previous studies by other groups [25, 26, 27, 28], we could demonstrate that AUD‐level drinking and alcohol withdrawal are indeed associated with increased neurotoxicity/axonal injury, as indicated by NFL values above the 90% percentile curve for healthy individuals provided by Harp et al. [47]. In this context, we could further show that the increase in NFL closely follows the clinical trajectory, with highest serum concentrations around the time of maximum withdrawal symptoms (i.e., on the third to fourth day of detoxification) as well as in the patients with the most severe drinking habits (as reflected by the positive correlation between AUDIT scores and NFL at T1 and T2). This demonstrates that NFL is not only able to replicate DTI‐based findings but can furthermore be used as a much more affordable and convenient marker for AUD‐associated axonal injury/WM damage throughout the course of detoxification treatment as well as in clinical studies aiming to reduce axonal injury/WM damage during alcohol withdrawal. Importantly, the fact that NFL levels significantly increased after the patients ceased to drink upon hospital admission suggests that the WM damage commonly observed in AUD patients cannot be entirely explained by the neurotoxicity of alcohol itself (if that was the case, NFL levels should have steadily declined from T1 to T4) but seems to be linked to the pathomechanisms of tolerance/withdrawal itself. Although our study does not provide direct evidence that glutamatergic excitotoxicity drives this process, we deem it to be a very plausible underlying mechanism that would be worth investigating further. Finally, the correlation of both thiamine and NFL levels with CANTAB performance underline the importance of managing both parameters as best as possible during AUD treatment and detoxification in order to minimize adverse short‐ and potential long‐term effects on cognitive‐behavioural functioning for the patients [48].

### 4.1 Limitations

Because we found a descriptive, but nonsignificant increase in NFL levels during the hungover state, and further given that the sequential analysis found more variance in the direction of effects in Study 2, we cannot exclude the possibility of very small/undetected increases in axonal injury/WM damage during alcohol hangover in at least some of the participants. Future studies with larger sample sizes and a more detailed assessment of potential stratification factors like age or immune fitness [49] could provide further insights.

Likewise, we did not assess any female participants in Studies 1 and 2 due to a lack of ethical permission to do so. Given the neurobiochemical, immunological and metabolic differences between the biological sexes, it would however be important to assess whether NFL levels in females are also not significantly affected by acute intoxication and hangover. Linked to this, a larger sample in Study 3 would have allowed to systematically investigate sex differences as well as differences in medications.

Lastly, it should be kept in mind that changes in NFL levels can reflect damage to both the peripheral and central nervous system [50], so that we cannot exclude the possibility that some of the NFL changes in the withdrawal sample might also (partly) reflect peripheral axonal damage.

Furthermore, future studies should ideally strive to obtain a more complete picture by investigating serum correlates of both WM and GM damage, for example, by also assessing the glial fibrillary acidic protein (GFAP) [51] or the tau protein [52, 53] in addition to NFL. Other correlates like oxidative stress should also be more closely controlled for [25].

## 5 Conclusions

Although there should be no doubt about the neurotoxicity of alcohol itself, our results clearly show that the widespread WM damage that has repeatedly been reported to be found in frequent drinkers and AUD patients might in relevant parts be caused by not by alcohol itself but by tolerance‐induced glutamatergic neurotoxicity instead (as this was the main factor distinguishing between intoxication/hangover and withdrawal). Given the observed increase of NFL during withdrawal and the association with cognitive and motor processes as well as AUD drinking habits, we would like to recommend and encourage the use of NFL as a clinical marker in alcohol detoxification. Future studies should further explore the link between alcohol tolerance/withdrawal, glutamatergic excitotoxicity, associated WM (and GM) damage and cognitive deficits in more detail. In case of accumulating evidence, clinical studies should then elucidate the possibility of reducing axonal injury/WM damage during withdrawal with NMDAR antagonists like carbamazepine, which is already approved for seizure management in AUD treatment [31] and might therefore be one of several promising options to help preserve cognitive functioning and control in AUD patients as best as possible.

## Author Contributions

**A.K.S., J.C.V., C.B.:** conceptualization. **A.K.S.:** methodology. **A.K.S., D.L., M.H., J.R.H., K.A.:** formal analysis. **A.K.S., M.H., J.R.H., D.L.:** investigation. **A.K.S., C.B., T.Z., K.A., M.P., E.B.:** resources. **A.K.S., D.L., M.H., J.R.H., K.A.:** data curation. **A.K.S.:** writing – original draft. **All authors:** writing – review and editing. **A.K.S.:** visualization. **A.K.S.:** supervision. **A.K.S.:** project administration. **A.K.S., C.B.:** funding acquisition.

## Funding

This study was supported by grants from the German Research Foundation (Deutsche Forschungsgemeinschaft, DFG) (TRR 265 B07) to A.K.S. and C.B., and a postdoctoral stipend from the Daimler and Benz Foundation (32‐10/19) to A.K.S. The funding parties had no role in study design; in the collection, analysis and interpretation of data; in the writing of the report; and in the decision to submit the article for publication.

## Ethics Statement

The study was conducted in accordance with the Declaration of Helsinki and its later amendments and approved by the local ethics committee of the TU Dresden, Germany (EK293082014). All study participants provided written consent before participating in the study and were informed about their right to withdraw from study participation at any moment. Participants were financially reimbursed (€60 in the acute intoxication effects study 1, €85 in the acute alcohol hangover study 2 and €50 in the acute withdrawal study 3).

## Conflicts of Interest

Dr. Verster reports personal fees from Eisai, personal fees from KNMP, personal fees from Med Solutions, personal fees from Red Bull and personal fees from Toast!, stock and travel support from Sen‐Jam Pharmaceutical, outside the submitted work. Dr. Verster is on the scientific advisory board of Sen‐Jam Pharmaceutical and Toast!. All other authors report no biomedical financial interests or potential conflicts of interest.

## Supporting information


**Table S1:** CANTAB task parameters used for behavioural analyses.
**Table S2:** Significant correlations between sober NFL levels, sociodemographic, alcohol‐related and sober CANTAB performance measures in Study 1 (intoxication).
**Table S3:** Significant correlations between sober NFL levels, sociodemographic, alcohol‐related and sober CANTAB performance measures in Study 2 (hangover).
**Table S4:** Significant correlations between NFL levels, sociodemographic, alcohol‐related and CANTAB performance measures in Study 3 (withdrawal).

## Data Availability

The data that support the findings of this study are available from the corresponding author upon reasonable request.
