# Supplementary material for: Effects of Binge Drinking and Alcohol Withdrawal on Serum Neurofilament Levels
Source: Addict Biol. 2026 Sep 15;31(9):e70183. doi: 10.1111/adb.70183 (PMC13578919; doi:10.1111/adb.70183)
Supplement: Supplementary file 1 [file ADB-31-e70183-s001.pdf]

Supplementary Information

Article title:	Effects of Binge Drinking and Alcohol Withdrawal on Serum Neurofilament Levels
Journal name:	Addiction Biology
Authors:	Ann-Kathrin Stock, Maximilian Pilhatsch, Katja Akgün, Joris C. Verster, Elisabeth Brückner, Mona Hofmann, Julian R. Hinz, Dušan A. Ladjević, Tjalf Ziemssen, Christian Beste
Corresponding author:	Ann-Kathrin.Stock@ukdd.de

METHODS

Study Design (additional details)

For the assessment of acute intoxication effects (study 1), we used a randomized interventional/experimental crossover study design, for which each participant was assessed once while sober and once while acutely intoxicated in an experimental / standardized manner. The appointment order was balanced across all participants so that half of the tested participants had their sober appointment before their intoxication appointment, whereas the other half had their intoxication appointment before their sober appointment. Both appointments were scheduled for a minimum of 48 hours and a maximum of 7 days apart from each other. All participants were asked to refrain from the use of caffeine, guanine, nicotine and all other sedative or stimulant substances within the last four hours prior to the start of each appointment. At the beginning of each appointment and at several time points during acute intoxication, breath alcohol concentration (BrAC) was measured using the breathalyzer "Alcotest 3000" (Drägerwerk, Lübeck, Germany). Participants were required to be sober (BrAC = 0.00 ‰) at the beginning of each appointment. The experimental procedure only resumed when sobriety was given.

For the assessment of acute alcohol hangover effects (study 2), we conducted a randomized interventional/experimental crossover studydesign, for which each participant was assessed once while sober and once while acutely hungover in an experimental / standardized manner. The appointment order was balanced across all participants so that half of the tested participants had their sober appointment before their intoxication and subsequent hangover appointments, whereas the other half had their intoxication and subsequent hangover appointments before their sober appointment. The sober and hangover appointments were scheduled for a minimum of 48 hours and a maximum of 7 days apart from each other. All participants were asked to refrain from the use of caffeine, guanine, nicotine and all other sedative or stimulant substances within the last four hours prior to the start of each appointment. At the beginning of each appointment and at several time points during acute intoxication, breath alcohol concentration (BrAC) was measured using the breathalyzer "Alcotest 3000" (Drägerwerk, Lübeck, Germany). Participants were required to be sober (BrAC = 0.00 ‰) at the beginning of each appointment. The experimental procedure only resumed when sobriety was given.

For the assessment of acute withdrawal effects (study 3), we used a non-interventional/observational longitudinal study design, for which each participant was assessed once at the beginning of their inpatient withdrawal treatment (T1), once at the maximum of their physical withdrawal symptoms (T2), once after the physical withdrawal symptoms had subsided (T3), and once before they were discharged from the clinics where they received treatment (T4). The T1 appointment (blood samples for NFL and thiamine, as well as CANTAB testing) took place on the first day of hospital admission (patients were instructed to maintain their normal drinking habits until the morning of admission). The T2 appointment (blood samples for NFL) took place 2-3 days after the T1 appointment, when physical withdrawal

symptoms tended to be the worst. The T3 appointment (blood samples for NFL, as well as CANTAB testing) took place on the first weekday after the physical withdrawal symptoms had subsided, which was usually between 5-7 days after hospital admission. The T4 appointment (blood samples for NFL, as well as CANTAB testing) took place on the last day before patients were discharged from inpatient treatment, which was usually 21 days after hospital admission.

Sample recruitment and inclusion criteria (additional details)

For study 1 (intoxication) and study 2 (hangover), healthy male participants aged 18-31 were recruited via online advertisements, flyers and posters using the same inclusion criteria as for previous hangover studies by our group (e.g., Opitz et al. [1]). Participants were required to have normal or corrected-to-normal vision, no prior history of physical, neurological or mental illness, and no acute or chronic medication intake that could influence liver or kidney function, or the central nervous system. Prior to inclusion in the study, applicants were screened in a telephone interview. This included the assessment of their physical and mental health with a semi-structured interview and their drinking pattern using the alcohol use disorders identification test (AUDIT)[2]. Applicants reporting alcohol-induced failure to perform daily routines and/or alcohol-related memory problems and/or binge drinking (i.e., consuming eight or more standard units of alcohol per occasion) “daily” or “almost daily”, were excluded because this would have indicated extensive problems associated with alcohol (ab)use and blurred the lines between studies 1/2 and study 3. In order to minimize the risk of intoxication-associated complications, we furthermore required all participants to have had practiced voluntary binge drinking at least once within the past 12 months. Further inclusion criteria on drinking patterns slightly varied between the intoxication and the hangover group, as the amount of alcohol administered was lower in the intoxication group than in the hangover study (please refer to the AUDIT-based inclusion criteria detailed in the following paragraph). The reason for this was that we only included participants who reported to have recently (within the last year) tolerated alcohol amounts comparable to those administered in their experimental study, and the absolute amount administered in the hangover study was higher than that administered in the intoxication study (due to differences in abstinence from food prior to the appointments - we had indeed planned to achieve comparable maximal intoxication levels in both experimental studies):For study 1 (intoxication), we included applicants who reported to binge drink at least once a year but no more than once a month, and who had an overall moderate/ non-high AUD risk pattern of alcohol use. This was defined by AUDIT scores between 1 and 15 points [2]. For study 2 (hangover), we included applicants who reported to binge drink at least once a month but no more than weekly / less than (almost) daily, and who had an overall moderate to harmful pattern of alcohol use, but without AUD diagnosis according to the ICD-10 criteria. This was defined by AUDIT scores between 2 and 19 points [2]. It should however be noted that we later decided to only include participants up to 15 points in both study 1 and study 2 to make them more comparable. Experienced psychologists screened all participants in study 1 and study 2 to make sure that none of them met the full diagnostic criteria required for an AUD diagnosis according to ICD-10.

For study 3 (withdrawal), participants seeking inpatient detoxification from alcohol aged 18-65 were screened and recruited by the medical staff of the University Clinics Dresden (Germany) and the Elblandklinikum Radebeul (Germany) when they applied for inpatient withdrawal treatment. Like for the other studies, patients were required to have normal or corrected-to-normal vision. They were

furthermore required to have a clinically confirmed diagnosis of AUD and alcohol had to be the primary substance of abuse. We did not include patients with Korsakoff syndrome or any other severe neurological, psychiatric, or developmental disorders that would have likely interfered with CANTAB task performance. Due to a high prevalence within AUD withdrawal patients, individuals with affective disorders linked to depression, anxiety/phobias, and PTSD were explicitly not excluded. Likewise, we allowed for the inclusion of patients with a history of seizures, as long as those were linked to past alcohol abuse and withdrawal. All participants were informed that their participation in the study would not alter their clinical treatment in any way other than the additional collection of blood samples and CANTAB performance testing.

All participants provided written informed consent prior the beginning of the experiment and received a financial reimbursement (60 € in study 1; 85 € in study 2; 50 € in study 3), or study credits (this was only applied in case of studies 1 and 2).

Sample size and sensitivity estimation

Based on observations from previous studies using the same study protocols as in study 1 and study 2 (e.g., Opitz et al. [1]), we expected the cognitive effects of acute alcohol intoxication to be larger than those of alcohol hangover.

We hence planned to assess enough participants in study 1 to be left with n=25 participants to analyze, which would yield a sensitivity for effects of $dz \geq 0.677$ (for one-sided paired t tests at an α error probability of 5% and a power of 95%, as estimated with G*Power [3]). To account for drop-outs and exclusions, we initially assessed n=30 participants in study 1 who also performed the CANTAB. Since there is no reliable information on whether and to what degree acute intoxication would alter NFL levels, we decided to additionally look at n=35 available blood samples from a previous intoxication study (assessed between September 2018 and April 2019) that had used the same study protocol, as well as inclusion and exclusion criteria [1,4,5].

We planned to assess enough participants in study 2 to be left with n=35 participants to analyze, which would yield a sensitivity for effects of $dz \geq 0.567$ (for one-sided paired t tests at an α error probability of 5% and a power of 95%, as estimated with G*Power [3]). To account for drop-outs and exclusions, we initially assessed n=43 participants in study 2 who also performed the CANTAB. Since there is no reliable information on whether and to what degree acute intoxication would alter NFL levels, we decided to additionally look at n=58 available blood samples from two previous hangover studies (assessed between February and March 2018, as well as May to June 2019) that had used the same study protocol, as well as inclusion and exclusion criteria [1,6–9].

In study 3, we had initially planned to assess n=45 patients, which would have allowed to detect effect sizes up to $f \geq 0.222$ (for a repeated-measures ANOVA with four time points at an α error probability of 5% and a power of 95%, as estimated with G*Power [3]). Due to the fact that the study data had to be collected during the COVID-19 pandemic (i.e., between March 2020 and December 2022), when studies were partly prohibited and the psychiatric ward was regularly closed for elective withdrawal treatment to make room for patients who needed care more urgently, we did unfortunately fall behind that goal and were only able to collect data from n=36 participants. Sensitivity analyses indicated that this sample size should still be sufficient to detect effect sizes of up to $f \geq 0.249$ (for a repeated-measures ANOVA with four time points at an α error probability of 5% and a power of 95%, as estimated with G*Power [3]).

Experimental alcohol administration

The experimental administration of a standardized amount of alcohol followed the protocol described as in Opitz et al. [1] (direct quotes in the following):

All participants were experimentally intoxicated in a controlled laboratory environment. For this purpose, we administered individual amounts of alcohol, which were calculated for each participant according to their estimated total body water (TBW). This was done by applying a version of the equation by Widmark (1932) and Watson et al. (1980) for males:

$$amount\ of\ alcohol\ in\ grams\ = \frac{TBW\ \times\ 1.055\ \times\ max.BrAC}{0.8}$$

where *TBW* is calculated as

$$TBW = 2.447 - (0.09516\ \times\ age) + (0.1074\ \times\ height) + (0.3362\ \times\ weight).$$

Group differences in experimental procedure and alcohol administration are detailed below.

Intoxication group

Intoxication appointments started on Friday or Saturday between 3 and 7 p.m.. Participants were asked to stop eating at least three hours prior to the intoxication appointment in order to induce the acute intoxication on a relatively empty stomach, as this reduces the resorption deficit, thus making rapid intoxication safer for the participants. Assuming a resorption deficit of 20 % on an empty stomach, an average BrAC of 1.2 ‰ can be achieved without exceeding the possible upper limit of 1.6 ‰ (which would have been reached at a resorption deficit of 0 %). To reach the goal of an average BrAC level of 1.2 ‰, we administered 1.98 grams of alcohol per liter of estimated TBW. This was converted into an individual amount of vodka (40 % alcohol by volume (ABV)) for each participant, which was diluted with the same amount of orange juice at room temperature. Mixtures of vodka and orange juice are considered the gold standard for orally induced acute intoxication, as this drink only contains very small amounts of congeners, thus reducing potential confounding factors, as well as the likelihood of next-day hangover [12]. The data sheet used to calculate individual alcohol amounts is provided as Supplementary Material. Participants were given 30 minutes to finish their individual beverage, and had another 30 minutes of waiting time before the experiment started. During the consumption and waiting time, participants could choose to either watch episodes of the television series “Shaun the Sheep” or the “Big Bang Theory” in order to standardize the entertainment during that time period and reduce the impact of variability of external factors during the waiting period on mood. Participants started the experimental task 30 minutes after the end of the alcohol consumption. BrAC was measured immediately before and after task completion. Additional BrAC measurements were also taken in between tasks. Unlimited access to tap water was provided during and after the experimental tasks at both the sober and intoxicated appointment.

Hangover group

To induce hangover symptoms, participants joined an intoxication appointment at our facilities on the night before the hungover appointment. This intoxication appointment was scheduled on a Friday or Saturday evening (starting at 8 p.m. and finishing at around 1:30 to 2 a.m.). Participants were asked to

partake on a full stomach to better cope with the administered alcohol amount and congener-rich alcohol type, as well as to reduce the likelihood of experiencing side effects like nausea, stomach pain and vomiting.

As the resorption deficit is higher on a full stomach (approx. 30-40 %), we administered 2.64 grams of alcohol per liter of estimated TBW. Given this assumed resorption deficit, participants were expected to achieve an average BrAC of 1.2-1.3 ‰ with only a very small likelihood of exceeding a BrAC of 1.6 ‰ (which would occur at a resorption deficit of 20 %, as typically observed on an empty stomach), and no possibility to exceed a max. BrAC of 2.0 ‰ (which would theoretically occur at a resorption deficit of 0 %). As an additional safety precaution, the consumption duration was extended to a minimum of 2 hours, which was controlled by the experimenters. Opposed to the vodka and orange juice mixture administered in the acute intoxication group, the hangover group was only served alcoholic beverages with a high congener content (e.g., brandy or red wine) as such beverages are more likely to provoke and lead to more intense hangover symptoms [12–14]. The individually calculated amount of alcohol was thus converted into amounts of brandy (36 % ABV) and/or red wine (9.5 % ABV). Both beverages were served by the experimenters in standardized portions of 200 mL red wine (15 gr of alcohol) or 50 mL brandy (14 g of alcohol) to ensure a constant quantity and speed of alcohol consumption across drinks. Experimenters furthermore recorded type and time of each issued drink for each individual participant. The data sheet used to calculate individual alcohol amounts and record consumption processes is provided as Supplementary Material. With each standardized drink, participants could choose between brandy and red wine, and whether to drink it pure, chilled on ice or mixed with decaffeinated coke, orange lemonade, or ginger ale. Snacks (chips and wine gums) and tap water were available at all times and their intake was not recorded. Given that smoking during alcohol consumption may increase the likelihood of occurrence and severity of hangover symptoms on the next morning [15], participants were allowed to smoke while drinking. BrAC was measured every 30 minutes after each participant's last sip of alcohol, that is 30, 60, 90 and 120 minutes after the end of consumption.

Hungover appointments started on Saturday or Sunday morning between 9 and 11 a.m. to slightly limit the amount of sleeping hours on the night before, as sleep duration seems to be negatively correlated with hangover severity [16] (in this context, it should however be noted that one of the main mechanisms via which binge-drinking affects sleep is a reduction in the subjective and objective quality of sleep [17]). To prevent a possible impact of residual ethanol in the blood, the hungover testing did not start until participants had reached BrAC readings of 0.00 ‰. Unlimited access to tap water was provided during and after the experimental task at both the sober and hungover appointment.

Assessment of cognitive-behavioral performance (additional details)

Part of the participants in study 1 (intoxication) and study 2 (hangover), as well as all of the participants in study 3 (withdrawal) were asked to complete several cognitive tests from the CANTAB® test battery (Cambridge Cognition) on 9.7'' iPad tablets (Apple, Inc). From the domain of "psychomotor speed", this included the Reaction Time test (RTI). From the domain of "attention", this included the Rapid Visual Information Processing test (RVP). From the domain of "executive function", this included the Spatial Working Memory test (SWM), the Stop Signal Task (SST), the Cambridge Gambling Task (CGT) and the Intra-Extra Dimensional Set Shift task (IED). The analyzed task parameters as well as the parameter descriptions provided by Cambridge Cognition are enlisted in *Table S-1* below.

Table S-1: CANTAB task parameters used for behavioral analyses

**Task**	**Parameter**	**Interpretation**
**Reaction Time (RTI)**		
RTI	FES	RTI Total Error Score (Five-Choice): The total number of trials where the subject made any form of response error. This measure is calculated through summing the inaccurate, incorrect location, omission and premature errors, alongside two other possible errors not individually output. These two additional errors are the use of multiple fingers and dragging a finger outside of a response box. Calculated across all assessment trials in which the stimulus could appear in any one of five locations. Please note that this outcome measure combines multiple, separate cognitive functions (i.e. an omission error is not psychologically the same as a premature error) and is therefore not a recommended measure for standard practice.
RTI	FMMT	RTI Mean Five-Choice Movement Time: The mean time taken for a subject to select the target stimulus after releasing the response button. Calculated across all correct, assessed trials in which the stimulus could appear in any one of five locations. Measured in milliseconds.
RTI	FMRT	RTI Mean Five-Choice Reaction Time: The mean duration it took for a subject to release the response button after the presentation of a target stimulus. Calculated across correct, assessed trials in which the stimulus could appear in any one of five locations. Measured in milliseconds.
RTI	SES	RTI Simple Error Score (all): The total number of trials where the subject made any form of response error. This measure is calculated through summing the inaccurate, omission and premature errors, alongside two other possible errors not individually output. These two additional errors are the use of multiple fingers and dragging a finger outside of a response box. Calculated across all assessment trials in which the stimulus could appear in one location only. Please note that this outcome measure combines multiple, separate cognitive functions (i.e. an omission error is not psychologically the same as a premature error) and is therefore not a recommended measure for standard practice.
RTI	SMMT	RTI Simple Mean Movement Time: The mean time taken for a subject to select the target stimulus after releasing the response button. Calculated across all correct, assessed trials in which the stimulus could appear in one location only. Measured in milliseconds.
RTI	SMRT	RTI Simple Mean Reaction Time: The mean time taken for a subject to release the response button after the presentation of a target stimulus. Calculated across correct, assessed trials in which the stimulus could appear in one location only. Measured in milliseconds.
**Rapid Visual Information Processing (RVP)**		
RVP	A	KEY: RVP A': A' (A prime) is the signal detection measure of a subject's sensitivity to the target sequence (string of three numbers), regardless of response tendency (the expected range is 0.00 to 1.00; bad to good). In essence, this metric is a measure of how good the subject is at detecting target sequences.

RVP	ML	RVP Mean Response Latency: The mean response latency of a correct response to a target sequence. Calculated across all assessed blocks.
RVP	PFAPercentile	KEY: RVP Probability of False Alarm: The number of sequence presentations that were false alarms divided by the number of sequence presentations that were false alarms plus the number of sequence presentations that were correct rejections: (False Alarms ÷ (False Alarms + Correct Rejections))
RVP	PH	RVP Probability of Hit: The number of target sequences during assessment blocks that were correctly responded to within the time allowed, divided by the number of target sequences during assessment blocks (Correct hits ÷ total number of sequences)
**Spatial Working Memory (SWM)**		
SWM	SPercentile	KEY: SWM Strategy (6-8 boxes): The number of times a subject begins a new search pattern from the same box they started with previously. If they always begin a search from the same starting point we infer that the subject is employing a planned strategy for finding the tokens. Therefore a low score indicates high strategy use (1 = they always begin the search from the same box), a high score indicates that they are beginning their searches from many different boxes. Calculated across assessed trials with 6 tokens or 8 tokens.
SWM	SX	SWM Strategy (6-12 boxes): The number of times a subject begins a new search pattern from the same box they started with previously. If they always begin a search from the same starting point we infer that the subject is employing a planned strategy for finding the tokens. Therefore a low score indicates high strategy use (1 = they always begin the search from the same box), a high score indicates that they are beginning their searches from many different boxes. Calculated across assessed trials with 6 tokens or more.
SWM	TE4	SWM Total errors 4 boxes: The number of times a box is selected that is certain not to contain a token and therefore should not have been visited by the subject, i.e. between errors + within errors - double errors. Calculated across all trials with 4 tokens only.
SWM	TE6	SWM Total errors 6 boxes: The number of times a box is selected that is certain not to contain a token and therefore should not have been visited by the subject, i.e. between errors + within errors - double errors. Calculated across all trials with 6 tokens only.
SWM	TE8	SWM Total errors 8 boxes: The number of times a box is selected that is certain not to contain a token and therefore should not have been visited by the subject, i.e. between errors + within errors - double errors. Calculated across all trials with 8 tokens only.
SWM	TE12	SWM Total errors 12 boxes: The number of times a box is selected that is certain not to contain a token and therefore should not have been visited by the subject, i.e. between errors + within errors - double errors. Calculated across all trials with 12 tokens only.
**Stop Signal Task (SST)**		
SST	SSRT	KEY: SST Stop Signal Reaction Time: The estimate of time where an individual can successfully inhibit their responses 50% of the time. This

		covert measurement is sampled from the length of time between the go stimulus and the stop stimulus at which the subject is able to successfully inhibit their response on 50% of the trials. We can infer that this is the time before which all actions become ballistic and the subject is no longer able to cancel their action selection.
**Cambridge Gambling Task (CGT)**		
CGT	DAVT	KEY: CGT Delay Aversion Total: Calculated by subtracting CGT Risk Taking for all trials from the ascending condition from CGT Risk Taking for all trials from the descending condition. A high score suggests high impulsivity. Allows for the dissociation of impulsivity from risk taking by determining whether a subject simply places a bet at the first opportunity.
CGT	DMAT	CGT Mean Decision Time Total Ascending: The mean time taken for a subject to select the colour on which to bet after presentation of the boxes. Calculated over all assessed trials from the ascending condition.
CGT	DMDT	CGT Mean Decision Time Total Descending: The mean time taken for the subject to select the colour on which to bet after presentation of the boxes. Calculated over all assessed trials from the descending condition.
CGT	DMMT	CGT Mean Decision Time Total Merged: The mean time taken for a subject to select the colour on which to bet after presentation of the boxes. Calculated over all assessed trials from both the ascending and descending conditions.
CGT	DMQAT	CGT Decision Making Quality Total Ascending: The proportion (0.0-1.0) of trials in the ascending condition where the subject chose the majority box colour, calculated by dividing the number of ascending trials where the subject chose the majority box colour by the total number of ascending trials. Calculated over all assessed trials from the ascending condition in which the number of boxes of each colour differed.
CGT	DMQDT	CGT Decision Making Quality Total Descending: The proportion (0.0-1.0) of trials in the descending condition where the subject chose the majority box colour, calculated by dividing the number of descending trials where the subject chose the majority box colour by the total number of descending trials. Calculated over all assessed trials from the descending condition in which the number of boxes of each colour differed.
CGT	DMQMT	KEY: CGT Decision Making Quality Total Merged: The proportion (0.0-1.0) of trials where the subject chose the majority box colour, calculated by dividing the number of trials where the subject chose the majority box colour by the total number of trials. Calculated over all assessed trials from both the ascending and descending conditions in which the number of boxes of each colour differed.
CGT	OPBAT	CGT Overall Proportion Bet Total Ascending: The mean proportion (0.0-1.0) of current points gambled by the subject,calculated by dividing the number of points gambled by the subject in a trial by the number of points they held at the start of that trial. Calculated over all

		assessed trials from the ascending condition, including those where the subject did not choose the majority box colour.
CGT	OPBDT	CGT Overall Proportion Bet Total Descending: The mean proportion (0.0-1.0) of current points gambled by the subject, calculated by dividing the number of points gambled by the subject in a trial by the number of points they held at the start of that trial. Calculated over all assessed trials from the descending condition, including those where the subject did not choose the majority box colour.
CGT	OPBMT	CGT Overall Proportion Bet Total Merged: The mean proportion (0.0-1.0) of current points gambled by the subject,calculated by dividing the number of points gambled by the subject in a trial by the number of points they held at the start of that trial. Calculated over all assessed trials from both the ascending and descending conditions, including those where the subject did not choose the majority box colour.
CGT	RAJAT	CGT Risk Adjustment Ascending: Risk adjustment is a measure of sensitivity to risk, based on the ability to modify choices when given information about the probability of different outcomes and to track the optimal outcome on each trial. The measure is calculated from the mean proportion of points that the subject chose to bet with, taking into account the number of coloured boxes in the majority, derived from all trials in the ascending condition.
CGT	RAJMT	KEY: CGT Risk Adjustment Merged: Risk adjustment is a measure of sensitivity to risk, based on the ability to modify choices when given information about the probability of different outcomes and to track the optimal outcome on each trial. The measure is calculated from the mean proportion of points that the subject chose to bet with, taking into account the number of coloured boxes in the majority.
CGT	RAJDT	CGT Risk Adjustment Descending: Risk adjustment is a measure of sensitivity to risk, based on the ability to modify choices when given information about the probability of different outcomes and to track the optimal outcome on each trial. The measure is calculated from the mean proportion of points that the subject chose to bet with, taking into account the number of coloured boxes in the majority, derived from all trials in the descending condition.
CGT	RTKAT	CGT Risk Taking Total Ascending: The mean proportion (0.0-1.0) of current points gambled by the subject, calculated by dividing the number of points gambled by the subject in a trial by the number of points they held at the start of that trial. Calculated over all assessed trials in the ascending condition in which the number of boxes of each colour differed and the subject chose the majority box colour.
CGT	RTKMT	CGT Risk Taking Total Merged: The mean proportion (0.0-1.0) of current points gambled by the subject, calculated by dividing the number of points gambled by the subject in a trial by the number of points they held at the start of that trial. Calculated over all assessed trials from both the ascending and descending conditions in which the number of boxes of each colour differed and the subject chose the majority box colour.

CGT	RTKDT	CGT Risk Taking Total Descending: The mean proportion (0.0-1.0) of current points gambled by the subject, calculated by dividing the number of points gambled by the subject in a trial by the number of points they held at the start of that trial. Calculated over all assessed trials in the descending condition in which the number of boxes of each colour differed and the subject chose the majority box colour.
CGT	TCB	CGT Total Blocks Completed: The total number of assessed blocks completed by the subject. This excludes trials which the subject terminated early.
**Intra-Extradimensional Set Shift (IED)**		
IED	ECS	IED Completed Stage Errors: The total number of errors made on stages that the subject successfully completed. This does not include the errors made on the stage (if any) on which the subject failed. Lower is better.
IED	EEDS	KEY: IED EDS Errors: The number of times that the subject failed to select the stimulus compatible with the current rule on the stage where the extra-dimensional shift occurs. This is a measure of the subject's ability to shift attentional set.
IED	EPRE	IED Pre-ED Errors: The number of times the subject chose the incorrect box for a stimulus on problems prior to the extra-dimensional shift. Lower is better.<br>This measure records the number of errors made prior to the extra-dimensional shift of the task. Errors are defined as instances when the subject fails to select the stimulus that is compatible with the current rule.
IED	TL	IED Total Latency: The sum of the subject's response latencies, measured in milliseconds, calculated across all assessed trials. The response time for a single trial is measured from the appearance of the stimuli, up to the time where the subject made their final choice for that trial.
IED	TT	IED Total Trials: This is the number of trials completed on all attempted stages. Lower is better.
IED	TTA	IED Total Trials (Adjusted): This is the number of trials completed on all attempted stages with an adjustment for any stages not reached. Lower is better.<br><br>Total Trials Adjusted = Total Trials (problems reached) + [(number of unreached problems) * (50)]<br><br>Subjects failing at any stage of the test will have had less opportunity to make choices (correct and erroneous). The adjustment is carried out to compensate for this missing data, and provide a more comparable error score to subjects completing all stages of the test.
IED	YCOST	IED Stages Completed: This is the total number of stages the subject completed successfully. There are nine stages to be completed. Higher is better.
IED	YERT	IED Total Errors: The total number of times that the subject chose a wrong stimulus - i.e. one incompatible with the current rule. Lower is

		better. This is a measure of the subject's efficiency in attempting the test.
IED	YERTA	KEY: IED Total Errors (Adjusted): The total number of times that the subject chose a wrong stimulus - i.e. one incompatible with the current rule, adjustment for every stage that was not reached. Lower is better.<br><br>Total Errors Adjusted = Total Errors (problems reached) + [(number of unreached problems) * (25)]<br><br>This is a measure of the subject's efficiency in attempting the test. Subjects failing at any stage of the test will have had less opportunity to make errors. The adjustment is carried out to compensate for this missing data, and provide a more comparable error score to subjects completing all stages of the test.
IED	YSTCO	IED Completed Stage Trials: This is the number of trials undertaken on all successfully completed stages. Lower is better.

All parameter descriptions are direct quotes from the information provided by the manufacturer.

RESULTS

Exclusions from the study samples after data collection / before data analyses

In study 1 (intoxication), we initially assessed n=65 male healthy participants. Out of those assessed, we excluded n = 14 for the following reasons: one or both blood samples could not be successfully drawn from n = 8 participants, sober NFL levels were above 10 and/or intoxicated NFL levels were above 15 for n=3 participants (for NFL norms for healthy individuals, please refer to Harp et al., [18]), n=1 participant stated to have hit his head hard 3 days prior to the sober appointment, n=1 participant was an outlier in terms of weight/administered alcohol and did not tolerate his dose well, and n=1 participant had a BDI of 13 or higher, indicating potential depression. Therefore, n = 51 participants entered the statistical analyses for neurofilament light chain (NFL) levels. Out of those, n = 24 had also performed the CANTAB test battery and were included in the behavioral analyses. Due to a lack of proper adherence to task instructions or understanding thereof, SWM data was missing for the intoxicated appointment of n=1 participant, RVP data was missing for the intoxicated appointment of n=1 participant, and IED data was missing for the intoxicated appointment of n=1 participant. Lastly, the BDI score is missing for n=1 participant. Those were treated as missing values, while the cases were kept in the analyses.

In study 2 (hangover), we initially assessed n=101 male healthy participants. Out of those assessed, we excluded n = 21 for the following reasons: one or both blood samples could not be drawn for n = 3 participants, sober NFL levels were above 10 and/or hungover NFL levels were above 15 in n= 2 participants, n=6 participants had an AUDIT score above 15, n=4 participants did not tolerate the intoxication dose well, n=1 participant had 4 or more alcoholic drinks on the night before the sober appointment, n=3 participants consumed more than 2 additional alcoholic drinks at home after the end of experimental intoxication, n=2 participants had a BDI score of 13 or higher. Therefore, n = 80 participants entered the statistical analyses for NFL levels. Of note, n=13 participants from study 2 had also participated in study 1 (participation in both studies was 1 year or more apart).

Out of the included n=80 participants, n = 35 had also performed the CANTAB test battery and were included in the behavioral analyses. Due to a lack of proper adherence to task instructions or understanding thereof, or due to unwellness, RVP data was missing for the sober appointment of n=1 participant. We furthermore deleted the RTI data of both appointments for n = 1 participant who seemed to have a slightly larger than average physiological tremor of unknown cause. Those were treated as missing values, while the cases were kept in the analyses.

In study 3 (withdrawal), we initially assessed n=36 healthy participants. Out of those assessed, we excluded n = 5 for the following reasons: n = 3 participants had NFL values above 45 (extreme outliers) during at least one of the assessed study appointments, n = 1 participant was suspected to potentially suffer from a mild case of Korsakoff syndrome, and n = 1 patient was in poor physical health due to comorbid anorexia nervosa. Therefore, n = 31 participants entered the statistical analyses for NFL levels.

Due du early discharge from the treatment program because of COVID-19 infections, other medical complications, relapses and patient-initiated treatment stops, there were n = 1 patient who only completed the T1 and T2 appointments, and n = 2 patients who only completed the T1, T2, and T3 appointments.

Due to a lack of proper adherence to task instructions or understanding thereof, or due to unwellness / strong withdrawal symptoms, RTI data was missing for the T1 appointment for n= 2 patients, RVP data was missing for the T1 appointment for n= 5 patients and for the T4 appointment for n = 2 patients, SWM data was missing for the T1 appointment for n= 3 patients, SST data was missing for the T1 appointment for n= 5 patients, and CGT data was missing for the T1 appointment for n= 5 patients and

for the T3 appointment for n = 1 patient. Lastly, serum thiamine values were missing for n = 7 patients, serum NFL values at T4 were missing in n = 2 patients, and questionnaire data were (partly) missing for n = 3 patients. All of those were treated as missing values, while the cases were kept in the analyses.

Correlation analyses for hypothesis 4 testing

Correlation analyses that were run for study 1: For the RTI task, we found several significant correlations suggesting fewer errors with higher NFL levels as well as fewer errors and faster responding with higher AUDIT scores. For the RVP task, we found a significant correlation suggesting faster responding with higher AUDIT scores.

Table S-2 details the significant correlations found between NFL levels, sociodemographic, alcohol consumption-related, and CANTAB performance measures in study 1.

Table S-2: Significant correlations between sober NFL levels, sociodemographic, alcohol-related, and sober CANTAB performance measures in study 1 (intoxication)

Variables	Significant correlation	Description of the effect
**General and drinking-related parameters**		
NFL sober * weight in kg	r = -0.427<br>p = 0.037<br>z = 0.296	Higher body weight was associated with lower NFL levels (i.e., less axonal injury / WM damage) during the sober state
**RTI**		
NFL sober * RTISES sober	r = -0.426<br>p = 0.038<br>z = -0.594	Higher NFL levels (i.e., more axonal injury / WM damage) during the sober state were associated with fewer overall errors in the simple condition of the RTI task when sober
AUDIT score * RTIFES sober	r = -0.500<br>p = 0.013<br>z = -0.454	Larger AUDIT scores (i.e., more risky drinking) were associated with a lower number of errors in the five choice condition of the RTI task when sober
AUDIT score * RTIFMMT sober	r = -0.458<br>p = 0.016<br>z = -0.530	Larger AUDIT scores (i.e., more risky drinking) were associated with faster mean movement times in the five choice condition of the RTI task when sober
AUDIT score * RTISMMT sober	r = -0.453<br>p = 0.026<br>z = -0.488	Larger AUDIT scores (i.e., more risky drinking) were associated with faster mean movement times in the simple condition of the RTI task when sober
**RVP**		
AUDIT score * RVPML sober	r = -0.441<br>p = 0.031<br>z = 0.473	Larger AUDIT scores (i.e., more risky drinking) were associated with a faster mean response latency in the RVP task when sober
**SWM**		
No significances		
**SST**		
No significances		
**CGT**		
No significances		

**IED**		
Not assessed in the sober state		

Correlation analyses that were run for study 2: For the RTI task, we found a significant correlation suggesting fewer errors with higher AUDIT scores. For the SWM task, we found a significant correlation suggesting more errors in one of the easier conditions with higher AUDIT scores. For the CGT task, we found a significant correlation suggesting that participants with higher NFL levels completed more blocks.

Table S-3 details the significant correlations found between NFL levels, sociodemographic, alcohol consumption-related, and CANTAB performance measures in study 2.

Table S-3: Significant correlations between sober NFL levels, sociodemographic, alcohol-related, and sober CANTAB performance measures in study 2 (hangover)

**Variables**	**Significant correlation**	**Description of the effect**
**General and drinking-related parameters**		
NFL sober * age in years	r = 0.368<br>p = 0.030<br>z = 0.386	Higher NFL levels (i.e., more axonal injury / WM damage) during the sober state were associated with an older age
**RTI**		
AUDIT score * RTIFES sober	r = -0.446<br>p = 0.008<br>z = -0.480	Larger AUDIT scores (i.e., more risky drinking) were associated with a lower number of errors in the five choice condition of the RTI task when sober
**RVP**		
No significances		
**SWM**		
AUDIT score * SWMTE6 sober	r = 0.355<br>p = 0.039<br>z = 0.371	Larger AUDIT scores (i.e., more risky drinking) were associated with a higher number of errors in the 6 box condition of the SWM task when sober
**SST**		
No significances		
**CGT**		
NFL sober * CGTTCB sober	r = 0.351<br>p = 0.039<br>z = 0.366	Higher NFL levels (i.e., more axonal injury / WM damage) during the sober state were associated with a higher number of blocks completed in the CGT task when sober
**IED**		
Not assessed in the sober state		

Correlation analyses that were run for study 3: For the RTI task, we found several significant correlations suggesting slower response times / motor responses in case of higher BrAC values / lower thiamine levels upon admission, lower overall AUD diagnostic criteria, as well as higher NFL levels at the

respective appointments. For the RVP task, we found significant correlations suggesting slower response latencies upon hospital admission in case of higher BrAC values / lower thiamine levels upon admission, but no relevant correlation of performance parameters with NFL levels at the respective appointments. For the SWM task, we found significant correlations between higher NFL levels during / after active withdrawal and a higher number of errors after active withdrawal. Yet, this result should be interpreted with ample caution as it was only found for one time point and almost exclusively in the easiest task condition. For the SST, we found significant correlation between higher NFL levels during active withdrawal and longer stop signal reaction time (SSRT) after active withdrawal. For the CGT, higher BrAC / lower serum thiamine on the day of hospital admission was associated with poorer decision making on the day of hospital admission and a few inconsistent effects on risk adjustment after acute withdrawal. Surprisingly, higher NFL levels were associated with lower risk taking and delay aversion on the respective appointments. For the IED task, higher BrAC on the day of hospital admission was associated with a lower number of errors made prior to the extra-dimensional shift of the IED task upon hospital discharge.

Table S-4 details the significant correlations found between NFL levels, sociodemographic, alcohol-related, and CANTAB performance measures in study 3.

Table S-4: Significant correlations between NFL levels, sociodemographic, alcohol-related, and CANTAB performance measures in study 3 (withdrawal)

**Variables**	**Significant correlation**	**Description of the effect**
**General and drinking-related parameters**		
BrAC T1 * NFL T4	r = 0.594<br>p = 0.001<br>z = 0.683	Larger BrAC values (i.e., higher intoxication) upon hospital admission were associated with higher NFL levels (i.e., more axonal injury / WM damage) upon hospital discharge
AUDIT score * NFL T1	r = 0.489<br>p = 0.008<br>z = 0.534	Larger AUDIT scores (i.e., more risky drinking) were associated with higher NFL levels (i.e., more axonal injury / WM damage) on the first day of withdrawal treatment
AUDIT score * NFL T2	r = 0.404<br>p = 0.033<br>z = 0.428	Larger AUDIT scores (i.e., more risky drinking) were associated with higher NFL levels (i.e., more axonal injury / WM damage) during the acute phase of withdrawal
AUDIT score * AUD criteria	r = 0.534<br>p = 0.003<br>z = 0.596	Larger AUDIT scores (i.e., more risky drinking) were associated with more fulfilled diagnostic DSM-V AUD criteria
**RTI**		
BrAC T1 * RTIFMRT T1	r = 0.534<br>p = 0.003<br>z = 0.595	Larger BrAC values (i.e., higher intoxication) upon hospital admission were associated with slower mean response times in the five choice condition of the RTI task upon hospital admission
BrAC T1 * RTISES T1 *	r = 0.431<br>p = 0.020<br>z = 0.461	Larger BrAC values (i.e., higher intoxication) upon hospital admission were associated with a higher number of errors in the simple / on choice condition of the RTI task upon hospital admission
BrAC T1 * RTISMRT T1	r = 0.405<br>p = 0.029<br>z = 0.430	Larger BrAC values (i.e., higher intoxication) upon hospital admission were associated with slower mean response times

		in the simple / one choice condition of the RTI task upon hospital admission
Thiamine T1 * RTIFMRT T3	r = -0.427<br>p = 0.042<br>z = -0.457	Higher serum thiamine levels upon hospital admission were associated with faster mean response times in the five choice condition of the RTI task after active withdrawal
Thiamine T1 * RTISMRT T3	r = -0.431<br>p = 0.040<br>z = -0.461	Higher serum thiamine levels upon hospital admission were associated with faster mean response times in the simple / one choice condition of the RTI task after active withdrawal
Thiamine T1 * RTISMRT T4	r = -0.444<br>p = 0.044<br>z = -0.477	Higher serum thiamine levels upon hospital admission were associated with faster mean response times in the simple / one choice condition of the RTI task upon hospital discharge
NFL T1 * RTISMRT T1	r = 0.393<br>p = 0.035<br>z = 0.416	Higher NFL levels (i.e., more axonal injury / WM damage) upon hospital admission were associated with slower mean response times in the simple / one choice condition of the RTI task upon hospital admission
NFL T2 * RTIFMRT T1	r = 0.447<br>p = 0.015<br>z = 0.481	Higher NFL levels (i.e., more axonal injury / WM damage) during active withdrawal were associated with slower mean response times in the five choice condition of the RTI task upon hospital admission
NFL T2 * RTISMRT T1	r = 0.482<br>p = 0.008<br>z = 0.526	Higher NFL levels (i.e., more axonal injury / WM damage) during active withdrawal were associated with slower mean response times in the simple / one choice condition of the RTI task upon hospital admission
NFL T3 * RTIFMMT T3	r = 0.375<br>p = 0.041<br>z = 0.394	Higher NFL levels (i.e., more axonal injury / WM damage) after active withdrawal were associated with slower mean movement times in the five choice of the RTI task after active withdrawal
NFL T3 * RTISMMT T3	r = 0.392<br>p = 0.032<br>z = 0.415	Higher NFL levels (i.e., more axonal injury / WM damage) after active withdrawal were associated with slower mean movement times in the simple / one choice condition of the RTI task after active withdrawal
AUD criteria * RTIFMMT T3	r = -0.405<br>p = 0.032<br>z = -0.430	A higher number of diagnostic DSM-V AUD criteria was associated with faster mean movement times in the five choice condition of the RTI task after active withdrawal
AUD criteria * RTIFMTT T3	r = -0.418<br>p = 0.027<br>z = -0.445	A higher number of diagnostic DSM-V AUD criteria was associated with faster mean response times in the five choice condition of the RTI task after active withdrawal
AUD criteria * RTIFES T4	r = 0.437<br>p = 0.023<br>z = 0.468	A higher number of diagnostic DSM-V AUD criteria was associated with a higher number of errors in the five choice condition of the RTI task upon hospital discharge
AUD criteria * RTIFMMT T4	r = -0.486<br>p = 0.010<br>z = -0.532	A higher number of diagnostic DSM-V AUD criteria was associated with faster mean movement times in the five choice condition of the RTI task upon hospital discharge
**RVP**		
BrAC T1 * RVPML T1	r = 0.587<br>p = 0.002<br>z = 0.659	Larger BrAC values (i.e., higher intoxication) upon hospital admission were associated with slower mean response latency in the RVT task upon hospital admission

Thiamine T1 * RVPML T1	r = -0.546<br>p = 0.013<br>z = -0.613	Higher serum thiamine levels upon hospital admission were associated with faster mean response latency in the RVT task upon hospital admission
AUDIT score * RVPPH T4	r = 0.454<br>p = 0.017<br>z = 0.490	Larger AUDIT scores (i.e., more risky drinking) were associated with a higher hit probability in the RVT task upon hospital discharge
**SWM**		
NFL T2 * SWMTE4 T3	r = 0.700<br>p < 0.001<br>z = 0.867	Higher NFL levels (i.e., more axonal injury / WM damage) during active withdrawal were associated with a higher number of errors in the easiest / 4 box condition of the SWM task after active withdrawal
NFL T3 * SWMTE4 T3	r = 0.546<br>p = 0.002<br>z = 0.612	Higher NFL levels (i.e., more axonal injury / WM damage) after active withdrawal were associated with a higher number of errors in the easiest / 4 box condition of the SWM task after active withdrawal
AUDIT score * SWMTE4 T3	r = 0.396<br>p = 0.037<br>z = 0.419	A higher AUDIT score (i.e., more risky drinking) was associated with a higher number of errors in the easiest / 4 box condition of the SWM task after active withdrawal
AUDIT score * SWMTE12 T3	r = -0.433<br>p = 0.022<br>z = -0.463	A higher AUDIT score (i.e., more risky drinking) was associated with a lower number of errors in the hardest / 12 box condition of the SWM task after active withdrawal
**SST**		
NFL T2 * SSRT T3	r = 0.407<br>p = 0.026<br>z = 0.432	Higher NFL levels (i.e., more axonal injury / WM damage) during active withdrawal were associated with a longer stop signal reaction time (SSRT) in the SST task after active withdrawal
**CGT**		
BrAC T1 * CGTDMQAT T1	r = -0.415<br>p = 0.035<br>z = -0.441	Larger BrAC values (i.e., higher intoxication) upon hospital admission were associated with a lower decision making quality in ascending trials of the CGT upon hospital admission
BrAC T1 * CGTDMQMT T1	r = -0.408<br>p = 0.039<br>z = -0.433	Larger BrAC values (i.e., higher intoxication) upon hospital admission were associated with a lower mean decision making quality in all trials of the CGT upon hospital admission
BrAC T1 * CGTRAJAT T3	r = 0.704<br>p < 0.001<br>z = 0.875	Larger BrAC values (i.e., higher intoxication) upon hospital admission were associated with better risk adjustment in ascending trials of the CGT after active withdrawal
BrAC T1 * CGTRAJDT T3	r = 0.624<br>p = 0.002<br>z = 0.732	Larger BrAC values (i.e., higher intoxication) upon hospital admission were associated with a better risk adjustment in descending trials of the CGT after active withdrawal
BrAC T1 * CGTRAJMT T3	r = -0.603<br>p = 0.003<br>z = -0.698	Larger BrAC values (i.e., higher intoxication) upon hospital admission were associated with a lower risk adjustment in all trials of the CGT after active withdrawal
BrAC T1 * CGTDAVT T4	r = -0.421<br>p = 0.029<br>z = -0.449	Larger BrAC values (i.e., higher intoxication) upon hospital admission were associated with a lower delay aversion in all trials of the CGT upon hospital discharge

Thiamine T1 * CGTDAVT T1	r = -0.502<br>p = 0.020<br>z = -0.553	Higher serum thiamine levels upon hospital admission were associated with a lower delay aversion in all trials of the CGT upon hospital admission
Thiamine T1 * CGTDMQAT T1	r = 0.462<br>p = 0.035<br>z = 0.499	Higher serum thiamine levels upon hospital admission were associated with a better decision making quality in ascending trials of the CGT upon hospital admission
Thiamine T1 * CGTDMQMT T1	r = 0.443<br>p = 0.044<br>z = 0.476	Higher serum thiamine levels upon hospital admission were associated with a better mean decision making quality in all trials of the CGT upon hospital admission
Thamine T1 * CGTRAJDT T3	r = 0.444<br>p = 0.023<br>z = 0.477	Higher serum thiamine levels upon hospital admission were associated with a better risk adjustment in descending trials of the CGT after active withdrawal
NFL T1 * CGTOPBDT T1	r = -0.639<br>p < 0.001<br>z = -0.756	Higher NFL levels (i.e., more axonal injury / WM damage) upon hospital admission were associated with lower proportion bets in descending trials of the CGT upon hospital admission
NFL T1 * CGTOPBMT T1	r = -0.569<br>p = 0.002<br>z = -0.647	Higher NFL levels (i.e., more axonal injury / WM damage) upon hospital admission were associated with lower proportion bets in all trials of the CGT upon hospital admission
NFL T1 * CGTRTKMT T1	r = -0.539<br>p = 0.001<br>z = -0.603	Higher NFL levels (i.e., more axonal injury / WM damage) upon hospital admission were associated with lower risk taking in all trials in the CGT upon hospital admission
NFL T1 * CGTRTKDT T1	r = -0.599<br>p = 0.001<br>z = -0.692	Higher NFL levels (i.e., more axonal injury / WM damage) upon hospital admission were associated with lower risk taking in descending trials in the CGT upon hospital admission
NFL T2 * CGTOPBDT T1	r = -0.683<br>p < 0.001<br>z = -0.835	Higher NFL levels (i.e., more axonal injury / WM damage) during active withdrawal were associated with lower proportion bets in descending trials of the CGT upon hospital admission
NFL T2 * CGTOPBMT T1	r = -0.591<br>p = 0.001<br>z = -0.679	Higher NFL levels (i.e., more axonal injury / WM damage) during active withdrawal were associated with lower proportion bets in all trials of the CGT upon hospital admission
NFL T2 * CGTRTKMT T1	r = -0.522<br>p = 0.003<br>z = -0.621	Higher NFL levels (i.e., more axonal injury / WM damage) during active withdrawal were associated with lower risk taking in all trials in the CGT upon hospital admission
NFL T2 * CGTDAVT T3	r = -0.464<br>p = 0.011<br>z = -0.502	Higher NFL levels (i.e., more axonal injury / WM damage) during active withdrawal were associated with a lower delay aversion in all trials of the CGT after active withdrawal
NFL T2 * CGTOPBDT T3	r = -0.468<br>p = 0.010<br>z = -0.508	Higher NFL levels (i.e., more axonal injury / WM damage) during active withdrawal were associated with lower proportion bets in descending trials of the CGT after active withdrawal
NFL T2 * CGTRTKDT T3	r = -0.418<br>p = 0.024<br>z = -0.445	Higher NFL levels (i.e., more axonal injury / WM damage) during active withdrawal were associated with lower risk taking in descending trials in the CGT after acute withdrawal
NFL T3 * CGTDAVT T3	r = -0.477<br>p = 0.009<br>z = -0.519	Higher NFL levels (i.e., more axonal injury / WM damage) after active withdrawal were associated with a lower delay aversion in all trials of the CGT after active withdrawal

NFL T3 * CGTOPBDT T3	r = -0.432<br>p = 0.019<br>z = -0.463	Higher NFL levels (i.e., more axonal injury / WM damage) after active withdrawal were associated with lower proportion bets in descending trials of the CGT upon hospital discharge
NFL T3 * CGTRAJAT T3	r = 0.465<br>p = 0.017<br>z = 0.503	Higher NFL levels (i.e., more axonal injury / WM damage) after active withdrawal were associated with better risk adjustment in ascending trials of the CGT after active withdrawal
NFL T3 * CGTRTKDT T3	r = -0.419<br>p = 0.024<br>z = -0.446	Higher NFL levels (i.e., more axonal injury / WM damage) after active withdrawal were associated with lower risk taking in descending trials in the CGT after active withdrawal
NFL T4 * CGTDAVT T4	r = -0.498<br>p = 0.010<br>z = -0.546	Higher NFL levels (i.e., more axonal injury / WM damage) upon hospital discharge were associated with a lower delay aversion in all trials of the CGT upon hospital discharge
NFL T4 * CGTRAJDT T4	r = 0.483<br>p = 0.012<br>z = 0.527	Higher NFL levels (i.e., more axonal injury / WM damage) upon hospital discharge were associated with better risk adjustment in descending trials of the CGT upon hospital discharge
NFL T4 * CGTRAJMT T4	r = 0.462<br>p = 0.017<br>z = 0.500	Higher NFL levels (i.e., more axonal injury / WM damage) upon hospital discharge were associated with better risk adjustment in all trials of the CGT upon hospital discharge
**IED (assessed at T4 only)**		
BrAC T1 * IEDEPRE T4	r = -0.449<br>p = 0.019<br>z = -0.484	Larger BrAC values (i.e., higher intoxication) upon hospital admission were associated with a lower number of errors made prior to the extra-dimensional shift of the IED task upon hospital discharge

Correlations between NFL values and performance parameters are only reported if both were collected on the same appointment (with the exception of T2 NFL values, for which correlations with T1 and T3 performance parameters is reported due to the lack of CANTAB data collection at T2).

REFERENCES

1. Opitz A, Ghin F, Hubert J, Verster JC, Beste C, Stock AK. Alcohol intoxication, but not hangover, differentially impairs learning and automatization of complex motor response sequences. *Sci Rep*. 2021;11(1):12539. doi:10.1038/s41598-021-90803-5
2. World Health Organization. AUDIT: the Alcohol Use Disorders Identification Test : guidelines for use in primary health care. *Screening and brief intervention for alcohol problems in primary care*. Published online 2001:WHO/MSD/MSB/01.6a. Accessed May 4, 2020. https://apps.who.int/iris/handle/10665/67205
3. Faul F, Erdfelder E, Lang AG, Buchner A. G*Power 3: A flexible statistical power analysis program for the social, behavioral, and biomedical sciences. *Behavior Research Methods*. 2007;39(2):175-191. doi:10.3758/BF03193146
4. Stock AK, Bensmann W, Zink N, Münchau A, Beste C. Automatic aspects of response selection remain unchanged during high-dose alcohol intoxication. *Addict Biol*. Published online December 10, 2019:e12852. doi:10.1111/adb.12852
5. Bensmann W, Zink N, Werner A, Beste C, Stock AK. Acute Alcohol Effects on Response Inhibition Depend on Response Automatization, but not on GABA or Glutamate Levels in the ACC and Striatum. *J Clin Med*. 2020;9(2). doi:10.3390/jcm9020481
6. Berghäuser J, Bensmann W, Zink N, Endrass T, Beste C, Stock AK. Alcohol Hangover Does Not Alter the Application of Model-Based and Model-Free Learning Strategies. *J Clin Med*. 2020;9(5):1453. doi:10.3390/jcm9051453
7. Opitz A, Beste C, Stock AK. Alcohol Hangover Differentially Modulates the Processing of Relevant and Irrelevant Information. *J Clin Med*. 2020;9(3). doi:10.3390/jcm9030778
8. Opitz A, Hubert J, Beste C, Stock AK. Alcohol Hangover Slightly Impairs Response Selection but not Response Inhibition. *J Clin Med*. 2019;8(9). doi:10.3390/jcm8091317
9. Zink N, Bensmann W, Beste C, Stock AK. Alcohol Hangover Increases Conflict Load via Faster Processing of Subliminal Information. *Front Hum Neurosci*. 2018;12:316. doi:10.3389/fnhum.2018.00316
10. Widmark EMP. *Die Theoretischen Grundlagen Und Die Praktische Verwendbarkeit Der Gerichtlich-Medizinischen Alkoholbestimmung*. Urban und Schwarzenberg; 1932.
11. Watson PE, Watson ID, Batt RD. Total body water volumes for adult males and females estimated from simple anthropometric measurements. *The American Journal of Clinical Nutrition*. 1980;33(1):1. doi:10.1093/ajcn/33.1.27
12. Rohsenow DJ, Howland J. The role of beverage congeners in hangover and other residual effects of alcohol intoxication: a review. *Curr Drug Abuse Rev*. 2010;3(2):76-79. doi:10.2174/1874473711003020076
13. Rohsenow DJ, Howland J, Arnedt JT, et al. Intoxication With Bourbon Versus Vodka: Effects on Hangover, Sleep, and Next-Day Neurocognitive Performance in Young Adults. *Alcoholism: Clinical and Experimental Research*. 2010;34(3):3. doi:10.1111/j.1530-0277.2009.01116.x
14. Verster JC. The alcohol hangover-a puzzling phenomenon. *Alcohol and Alcoholism*. 2008;43(2):2. doi:10.1093/alcalc/agm163
15. Jackson KM, Rohsenow DJ, Piasecki TM, Howland J, Richardson AE. Role of Tobacco Smoking in Hangover Symptoms Among University Students. *J Stud Alcohol Drugs*. 2013;74(1):1. doi:10.15288/jsad.2013.74.41
16. van Schrojenstein Lantman M, van de Loo A, Mackus M, Verster J. Development of a Definition for the Alcohol Hangover: Consumer Descriptions and Expert Consensus. *CDAR*. 2017;9(2):2. doi:10.2174/1874473710666170216125822
17. Devenney LE, Coyle KB, Roth T, Verster JC. Sleep after Heavy Alcohol Consumption and Physical Activity Levels during Alcohol Hangover. *J Clin Med*. 2019;8(5). doi:10.3390/jcm8050752
18. Harp C, Thanei GA, Jia X, et al. Development of an age-adjusted model for blood neurofilament light chain. *Ann Clin Transl Neurol*. 2022;9(4):444-453. doi:10.1002/acn3.51524
